# Agent Repurposing for the Treatment of Advanced Stage Diffuse Large B-Cell Lymphoma Based on Gene Expression and Network Perturbation Analysis

**DOI:** 10.3389/fgene.2021.756784

**Published:** 2021-10-14

**Authors:** Chenxi Xiang, Huimin Ni, Zhina Wang, Binbin Ji, Bo Wang, Xiaoli Shi, Wanna Wu, Nian Liu, Ying Gu, Dongshen Ma, Hui Liu

**Affiliations:** ^1^ Department of Pathology, The Affiliated Hospital of Xuzhou Medical University, Xuzhou, China; ^2^ Department of Pathology, Xuzhou Medical University, Xuzhou, China; ^3^ Department of Oncology, Emergency General Hospital, Beijing, China; ^4^ Genies Beijing Co., Ltd., Beijing, China; ^5^ Qingdao Geneis Institute of Big Data Mining and Precision Medicine, Qingdao, China

**Keywords:** diffuse large B-cell lymphoma, drug repurposing, differentially expressed genes, differential module analysis, key driver analysis

## Abstract

Over 50% of diffuse large B-cell lymphoma (DLBCL) patients are diagnosed at an advanced stage. Although there are a few therapeutic strategies for DLBCL, most of them are more effective in limited-stage cancer patients. The prognosis of patients with advanced-stage DLBCL is usually poor with frequent recurrence and metastasis. In this study, we aimed to identify gene expression and network differences between limited- and advanced-stage DLBCL patients, with the goal of identifying potential agents that could be used to relieve the severity of DLBCL. Specifically, RNA sequencing data of DLBCL patients at different clinical stages were collected from the cancer genome atlas (TCGA). Differentially expressed genes were identified using DESeq2, and then, weighted gene correlation network analysis (WGCNA) and differential module analysis were performed to find variations between different stages. In addition, important genes were extracted by key driver analysis, and potential agents for DLBCL were identified according to gene-expression perturbations and the Crowd Extracted Expression of Differential Signatures (CREEDS) drug signature database. As a result, 20 up-regulated and 73 down-regulated genes were identified and 79 gene co-expression modules were found using WGCNA, among which, the thistle1 module was highly related to the clinical stage of DLBCL. KEGG pathway and GO enrichment analyses of genes in the thistle1 module indicated that DLBCL progression was mainly related to the NOD-like receptor signaling pathway, neutrophil activation, secretory granule membrane, and carboxylic acid binding. A total of 47 key drivers were identified through key driver analysis with 11 up-regulated key driver genes and 36 down-regulated key diver genes in advanced-stage DLBCL patients. Five genes (*MMP1*, *RAB6C*, *ACCSL*, *RGS21* and *MOCOS*) appeared as hub genes, being closely related to the occurrence and development of DLBCL. Finally, both differentially expressed genes and key driver genes were subjected to CREEDS analysis, and 10 potential agents were predicted to have the potential for application in advanced-stage DLBCL patients. In conclusion, we propose a novel pipeline to utilize perturbed gene-expression signatures during DLBCL progression for identifying agents, and we successfully utilized this approach to generate a list of promising compounds.

## Introduction

Diffuse large B-cell lymphoma (DLBCL) is the most commonly diagnosed non-Hodgkin lymphoma (NHL), representing approximately 25% of new NHL cases each year in the United States (Liu and Barta, 2019). In practice, about one half of DLBCL patients presented with advanced-stage disease (Prakash et al., 2012), featuring bulky tumor burden and poor patient response to treatment. According to published data, advanced-stage DLBCL (stage I/II and stage III/IV) may be both biologically and clinically different from limited-stage DLBCL cases (stage I and II). For example, advanced-stage DLBCL patients were more likely to express higher levels of CD30 (Rodrigues-Fernandes et al., 2021) and CD25 (Oka et al., 2020), both of which are biomarkers of B-cell activation. In addition, advanced-stage DLBCL was also shown to be associated with a higher immune-inflammation index (Wang et al., 2021) and an increased level of lymphopenia at diagnosis (Shin et al., 2020), highlighting its deteriorating immune regulation. Green and Johnson et al. reported there were a few biological factors known to adversely impact the prognosis of DLBCL patients, including the cell-of-origin, co-expression of MYC/BCL2 and co-occurrence of *MYC* and *BCL2/BCL6* rearrangements failed to predict poorer prognosis in limited stage DLBCL(Green et al., 2012; Johnson et al., 2012). Ajay, Major *et al* reported that stage I and II DLBCL cases had a slightly increased risk of secondary primary malignancies after DLBCL treatment in long-term follow-up (>20 years) (Major et al., 2020). Comparing with limited stage DLBCL, advanced-stage DLBCL patients were more likely to benefit from intensified radiotherapy (Hoiland et al., 2020; Freeman et al., 2021). Also, the pattern of late disease relapses observed in advanced stage DLBCL cases was different from that of limited-stage cases, further corroborating that limited and advanced stage DLBCL were biologically heterogeneous (Hoiland et al., 2020). All of these observations prompted us to treat advanced- and limited-stage DLBCL with different strategies, better tailoring for their specific biological and clinical characteristics.

However, there is limited knowledge regarding the genomic and transcriptomic differences between limited- and advanced-stage DLBCL. Two previous large analyses exploring the genetic landscape of DLBCL were not intended to compare the limited and advanced stages of the disease (Reddy et al., 2017; Schmitz et al., 2018). Moreover, at the single gene or single locus level, advanced- and limited-stage DLBCL may also be different in terms of their altered gene regulation and regulatory/co-expression networks, which was confirmed in other clinical comparisons such as cancer vs normal tissue (Zhang et al., 2018; Xu et al., 2019) and young vs old (Yang et al., 2015; Yang et al., 2016b).

Although frontline chemoimmunotherapies have been shown to cure up to 60% of patients with advanced-stage disease, with a clear plateau in progression-free survival (PFS) and rare relapses beyond 5 years (Coiffier et al., 2010), there still is a fraction of patients who are subject to relapse and have tumors that are refractory to treatment (Coiffier et al., 2010), highlighting the heterogeneity of advanced DLBCL. Thus, it is critical to develop new drugs for improving the treatment of advanced-stage DLBCL, so that it might be effectively treated by using existing treatment strategies as limited-stage DLBCL patients are. However, the development of a novel drug is usually costly and time-consuming (Liu et al., 2020; Yang et al., 2020) and highlights the need for effective drug repositioning strategies. There are many computer-based drug repositioning methods that have been used for cancers (Xu et al., 2019; Liu et al., 2020) and other diseases, such as Coronavirus disease 2019 (COVID-19) (Tang et al., 2020; Li et al., 2021).

In this study, we propose a new strategy for identifying new agents that have the potential to specifically target advanced-stage DLBCL. In general, we retrieved advanced-stage DLBCL-specific expressed genes by comparing the transcriptome of advanced-stage disease with that of limited-stage DLBCL. These differentially expressed genes (DEGs) were then subjected to weighted gene correlation network analysis (WGCNA) to discover the co-expression modules that may contribute to the progression of this disease. Finally, potential personal agents were obtained from the Crowd Extracted Expression of Differential Signatures (CREEDS) based on the down-regulation and up-regulation of genes (see Materials and methods for details). We aimed to specifically reveal the transcriptomic scenario occurring in advanced-stage DLBCL and to elucidate the genes that were most likely contributing to disease progression. Based on this knowledge, we then identified some potential agents for the treatment of advanced-stage DLBCL in future clinical practice.

## Materials and Methods

### Data Collection

RNA sequencing data from patients with DLBCL cancer were collected from the cancer genome atlas (TCGA). Based on the imaging results, including computed tomography (CT) scans, magnetic resonance imaging (MRI) or positron emission tomography (PET) scanning, patients were divided into four stages (I–IV) according to the Ann Arbor system (Heidelberg, 2020).

### Differential Gene Expression Analysis Between Samples at Different Stages

An expression matrix of 42 patients and their group information (stage I/II or III/IV) were used as the input for DEG discovery. DEGs between samples at stage I/II and stage III/IV were obtained using DESeq2 (Love et al., 2014) using log_2_ |fold change| ≧ 1 and a *p* value ≦ 0.05.

### Survival Analysis

After identifying DEGs, we performed survival analysis on these genes for all of the patients. Next, Kaplan-Meier (Bland and Altman, 1998) survival estimation was used for all differentially expressed genes to identify genes correlated with overall survival. Kaplan- Meier arranged the survival time in descending order, at each death node, it estimated the proportion of the observed values that survived for a certain period of time under the same circumstances, which could intuitively show the survival and mortality rates of two or more groups. The R packages survival and survminer were used for survival analysis and curve plotting, respectively.

### Weighted Gene Correlation Network Analysis

The WGCNA package in R (Peter and Horvath, 2008) was used to construct a co-expression network. For this step, we randomly picked 400 genes from the stage III/IV patients to generate a topological overlap matrix since the gene number was too large to perform this analysis using all of the genes. For the constructed gene network to conform to a scale-free distribution, a soft threshold was used to select the appropriate β after removing outliers. Finally, the soft threshold was set to 10. Then, genes were clustered by hierarchical clustering, and the tree was cut into different modules using a dynamic cutting algorithm, in which genes were highly correlated. Furthermore, we calculated the Pearson correlation coefficient between different modules and clinical stage and used this Pearson correlation coefficient to judge the relationship between the module and clinical stage. Finally, significant modules closely related to the occurrence and development of DLBCL were identified for follow-up analysis.

### Functional and Pathway Enrichment Analyses

KEGG pathway (Ogata et al., 1999) analysis and Gene Ontology (GO) analysis (Botstein et al., 2000), including biological process (BP), cellular composition (CC) and molecular function (MF), were performed on the genes in the module identified by WGCNA to understand the biological significance of the progression of DLBCL. The R package clusterProfiler (Yu et al., 2012) was used in the process of enrichment analysis to analyze the functions of the genes from these modules.

### Key Driver Analysis

For key driver analysis, we used up- or down-regulated genes separately as inputs to identify key drivers. Key driver analysis (Yang et al., 2016a) (KDA) was used to identify hub genes, and protein actions v11.0 was used as a reference protein–protein interaction network (Szklarczyk et al., 2021). Parameters were set as follows: nlayerExpansion was set to 1, nlayerSearch was set to 6 and enrichedNodesPercentCut was set to −1. A *p* value_whole ≦ 0.05 was used to filter out key drivers. The hub genes were of great significance in terms of the occurrence and development of DLBCL.

### Drug Discovery

CREEDS includes single gene perturbation signatures, as well as disease and drug perturbation signatures, and it can be used to identify the relationship between gene, disease and drug (Gillies et al., 2016). CREEDS is composed of single-drug perturbation-induced gene expression signatures. Utilizing this database, agents that can reverse the behavior of up/down-regulated genes can be discovered, and the best matched agents are reported. We used this tool for drug discovery for advanced-stage DLBCL. In this work, we combined differentially expressed genes and key driver genes as a new gene set to discover new agents related to advanced-stage DLBCL.

## Results

### A Brief Study Design of Drug Repurposing

**FIGURE 1:**
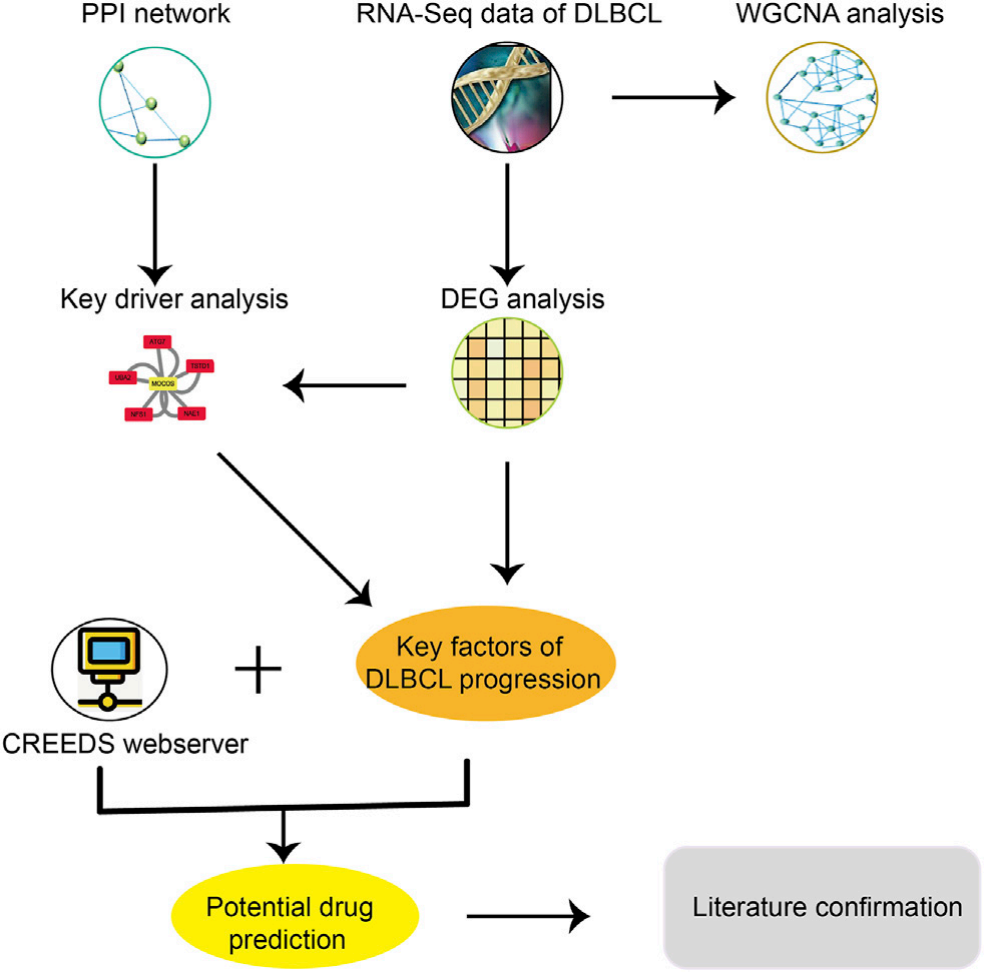
A brief study design for drug repurposing, including these major steps: 1) Download and organize the RNA-seq data and clinical information of DLBCL from TCGA; 2) Got key factors of DLBCL progression through DEG analysis, key driver analysis and WGCNA analysis; 3) Potential drug prediction through CREEDs; 4) Literature confirmation.

For the purpose of specifically developing new agents that could be utilized in combination with R- CHOP backbones to treat advanced stage DLBCL patients, we proposed a new method of drug repurposing based on gene expression and network perturbation (Figure 1). In order to identify key factors for DLBCL progression, WGCNA and DEG, differential module (DM) and key driver (KD) analyses were performed. Then, the key factors of DLBCL progression and drug perturbation signature were used to predict potential agents for the treatment of advanced stage DLBCL. Finally, some previous studies were reviewed to demonstrate the effectiveness of the newly identified agents.

### Patient Characteristics

The clinical characteristics of DLBCL cancer patients collected from TCGA are presented in Table 1, including 25 patients at clinical stage I/II and 17 patients at clinical stage III/IV. It was more likely to occur in elder patients and involve extranodal sites or organs. Patients of advanced stage disease also tended to have B symptoms. No gender preference was observed in this group of patients and all patients received no treatment before resection of tumors.

**TABLE 1:** Summary of general clinical information of DLBCL cases in TCGA.

		Limited stage	Advanced stage	$\chi^{2}$	*P*
Gender	Male	9	10	0.006	0.938
	Female	16	7		
Age	≥60	6	10	5.203	0.023
	<60	19	7		
Extranodal disease	Yes	8	11	4.369	0.037
	No	17	6		
B symptoms	Yes	1	9	13.36	0.000
	No	24	8		

### Identification of DEGs and Survival Analysis

**FIGURE 2:**
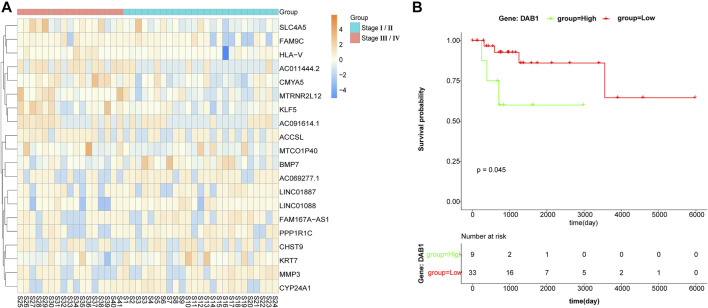
Analysis of differentially expressed genes. **(A)** Heat map of the top 10 differentially expressed genes. The *x*-axis represents different samples from TCGA, blue indicates samples at limited stage (stage I/II) and red indicates samples at advanced stage (stage III/IV). The *y*-axis represents differentially expressed genes. **(B)** Survival curve of the association between the expression levels of *DAB1* and survival time after diagnosis with DLBCL.

After collecting data from TCGA, DEGs were obtained using DESeq2, by comparing the transcriptome of advanced stage DLBCL with limited stage DLBCL. Of the 93 DEGs that were identified with a log_2_ |fold change| ≧ 1 and a *p* value ≦ 0.05, 20 genes were up-regulated and 73 genes were down-regulated in advanced DLBCL. The top 10 genes that were differently expressed between advanced and limited stage DLBCL are shown in Figure 2A.

We aimed to evaluate whether this set of differentially expressed genes could define a group of patients with poorer prognosis. We dichotomized 42 DLBCL cases into either the high expression group or the low expression group as per the mean expression level of each DEG. In addition, the Kaplan-Meier survival estimation method was used to evaluate all DEGs to study the relationship between gene expression and overall survival. Through this Kaplan-Meier survival estimation analysis, we found that *DAB1* was negatively correlated with overall survival, while other DEGs were not correlated with overall survival.

### Weighted Gene Correlation Network Analysis and Differential Model Analysis

**FIGURE 3:**
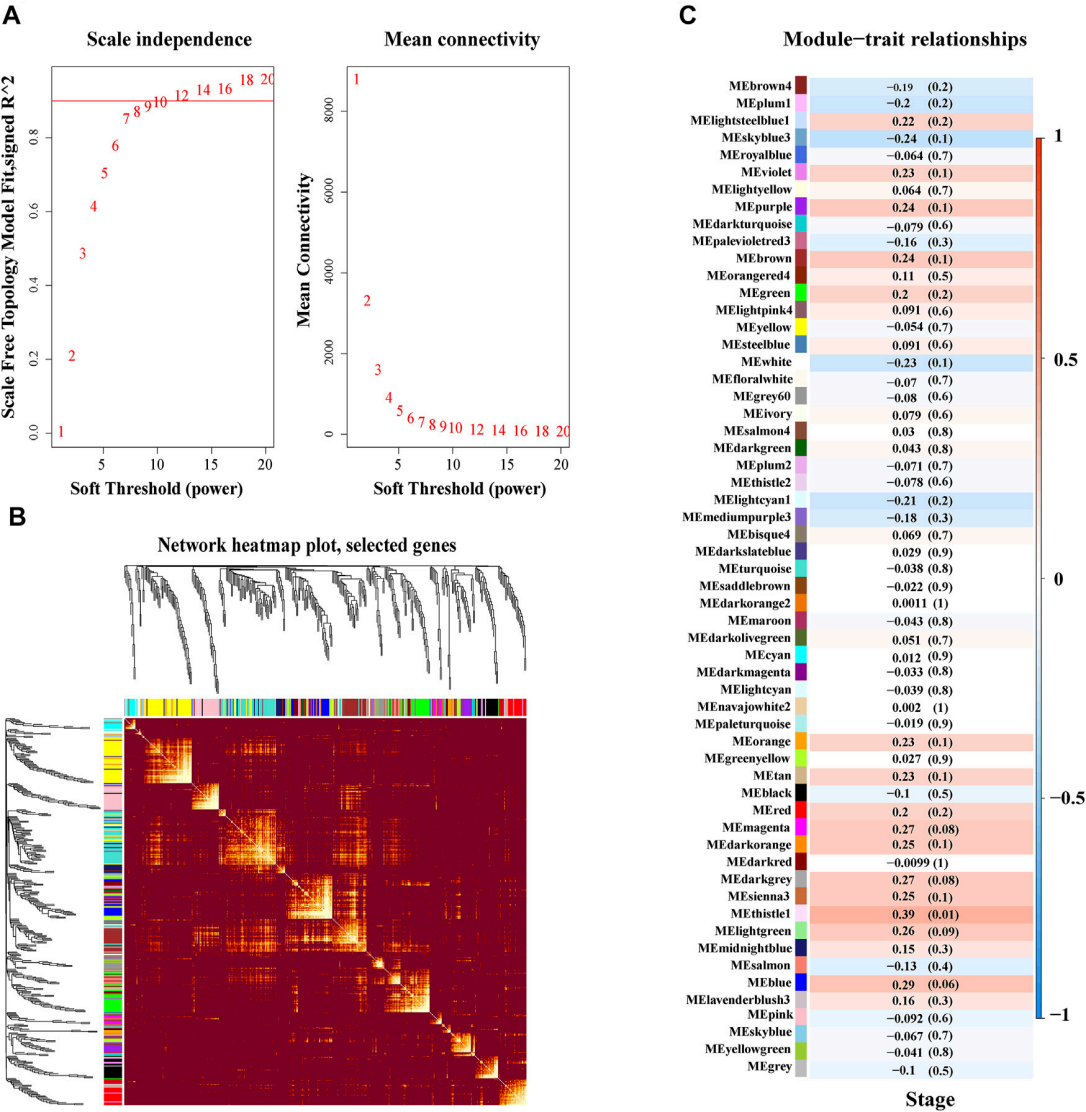
Weighted co-expression and key module identification associated with clinical DLBCL stage. **(A)** Determination of soft threshold in WGCNA. **(B)** Topological overlapping WGCNA heat map. **(C)** The relationship between modules and clinical traits. Pearson correlation coefficient was used to calculate the correlation degree between each module and trait.

WGCNA, based on a scale-free network to analyze genes according to their expression patterns, was used to cluster highly related genes into one module. As can be seen from Figure 3A, the soft threshold value was set at 10 to build this scale-free network. Next, 79 gene modules were identified by hierarchical clustering and dynamic branch cutting, and each module was assigned a unique color identifier (Supplementary Figure S4). We then selected a portion of these genes to construct a topological overlapping heat map, shown in Figure 3B. Through differential module analysis, we found that the thistle1 module was most relevant to advance stage of DLBCL in this dataset (Figure 3C).

### Functional and Pathway Enrichment Analysis of the thistle1 Module

**FIGURE 4:**
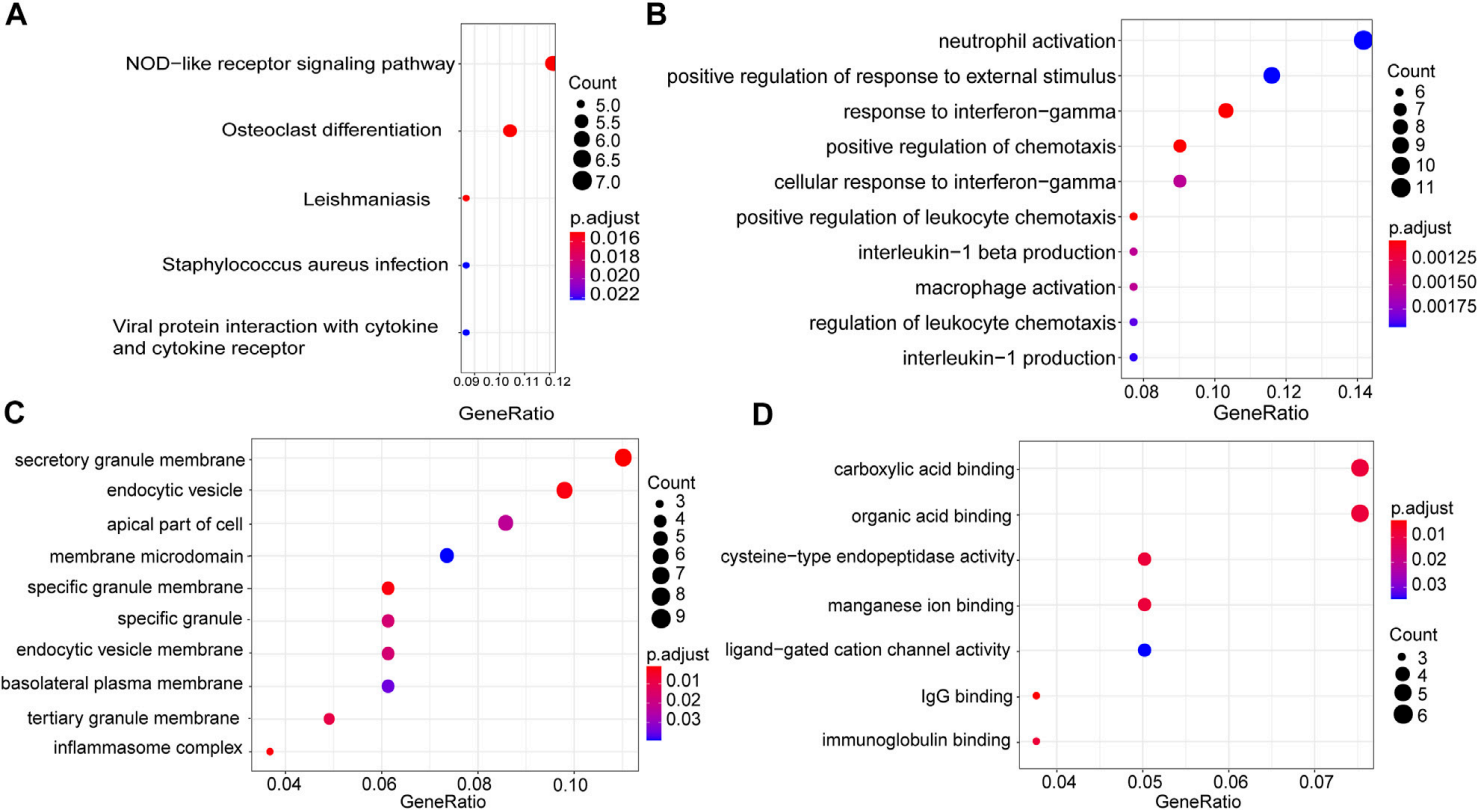
Pathway and functional enrichment analysis of genes in the thistle1 module. **(A)** KEGG pathway analysis. **(B)** GO enrichment for biological process. **(C)** GO enrichment for cellular composition. **(D)** GO enrichment for molecular function. The *x*-axes are the ratio of genes, and the *y*-axes are the GO terms.

In order to understand the causes of DLBCL deterioration from the biological level, we analysed the genes in the thistle1 module using KEGG pathway and GO enrichment analysis. KEGG pathway analysis results indicated that the development of DLBCL was very strongly correlated to the NOD-like receptor signalling pathway, osteoclast differentiation, leishmaniasis, *Staphylococcus aureus* infection and viral protein interaction with cytokine and cytokine receptor (Figure 4A). Furthermore, GO enrichment was performed based on three aspects: BP, CC and MF. In the BP analysis, we found that the genes in the thistle1 module were mainly related to neutrophil activation, positive regulation of response to external stimulus and response to interferon−gamma (Figure 4B). In addition, in the CC analysis, the genes in the thistle1 module were related to secretory granule membrane, endocytic vesicle and apical part of cell (Figure 4C). Moreover, the genes in the thistle1 module were mainly enriched in 7 MFs, including carboxylic acid binding, organic acid binding, cysteine−type endopeptidase activity, manganese ion binding, ligand−gated cation channel activity, immunoglobulin G (IgG) binding and immunoglobulin binding (Figure 4D).

### Hub Genes Identified Through Key Driver Analysis

**FIGURE 5:**
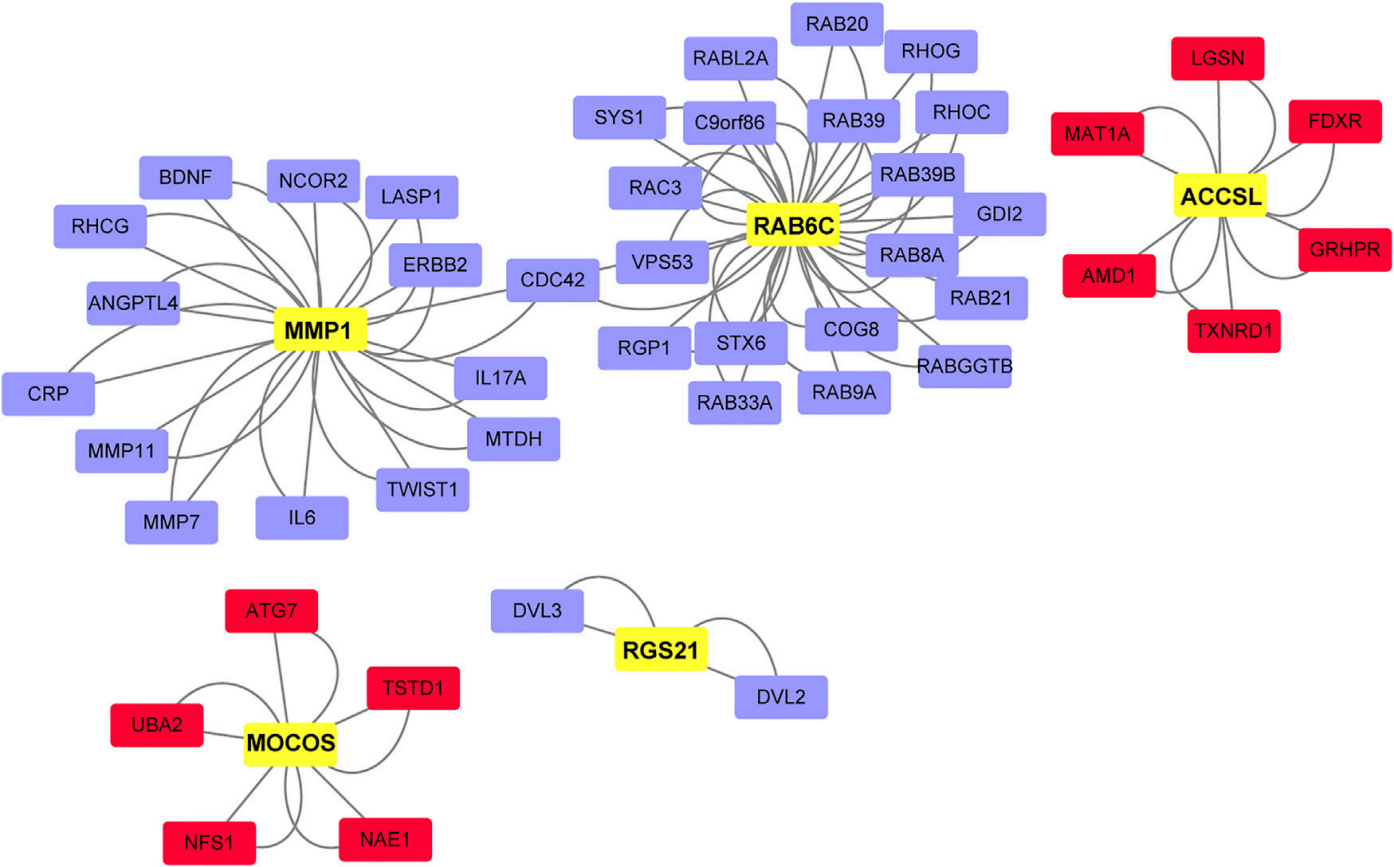
Network of key DLBCL drivers and hub genes. Red, key drivers from up-regulated gene set in advanced-stage samples. Blue, key drivers from down-regulated gene set in advanced-stage samples. Yellow, hub genes.

A total of 47 key drivers were identified through key driver analysis, with 11 up-regulated key driver genes and 36 down-regulated key diver genes being diagnostic of advanced-stage DLBCL relative to limited-stage DLBCL. Then, five hub genes were identified from key drivers as shown in Figure 5, which were most related to the occurrence and development of DLBCL. *MMP1* (Rosas et al., 2008), also known as matrix metalloproteinase-1, encodes a protein of 469 amino acid residues and is a kind of photolytic enzyme closely related to tumor genesis, invasion and metastasis. *Rab6c* (Young et al., 2010) is a member of the RAS family. Its mutation can affect the balance of Ras-GTP and cause malignant transformation of cells. Gene ontology annotations for 1-Aminocyclopropane-1-Carboxylate Synthase Homolog (Inactive) Like (*ACCSL*) (Chen and Karampinos, 2020) include pyridoxal phosphate binding. Dysregulation of gene levels of molybdenum cofactor sulfurase (*MOCOS*) (Kurzawski et al., 2012) can lead to cell disorders. Studies have demonstrated that this gene can be used as a key detection gene for kidney genetic diseases. *RGS21* (Von Buchholtz et al., 2004), a new member of the regulator of G protein signaling (RGS) protein family. It can inhibit signal transduction by increasing GTPase activity.

### Agent Screening

Potential personal agents associated with DLBCL were identified according to the differences between differential genes and drug signaling. Approximately 10 potential agents were selected according to their drug perturbation-induced gene expression signatures, and detailed information on these agents is presented in Table 2, including the type, drug/small molecule, possible effect and evidence for activity. The top five agents could reverse the expression of down-regulated genes, and the remaining agents could reverse the expression of up-regulated genes. In other words, after treatment with these drugs, gene expression levels may return to normal. The top five agents that may reverse down-regulated gene expression are formaldehyde, ethanol, dibutyl phthalate, paclitaxel, and prednisolone. Ethanol (EtOH) is similar to pharmacological mTOR inhibitors and has been shown to inhibit the mTOR signaling pathway. Mazan et al. studied the influence of EtOH on the mTOR signaling pathway and explored the translational group analysis of downstream effects of EtOH in DLBCL, and the results showed that EtOH partially inhibited mTOR signaling and protein translation (Mazan-Mamczarz et al., 2015). In a previous study, newly diagnosed DLBCL patients treated with rituximab, cyclophosphamide, doxorubicin, vincristine, and prednisolone (R-CHOP) were evaluated for their clinical characteristics, therapeutic efficacy and patient survival, and DLBCL patients treated with R-CHOP had better survival than other patients (Hong et al., 2011). Ohe et al. also reported a case of DLBCL successfully treated with prednisolone (Ohe et al., 2012). The top five agents that may reverse up-regulated gene expression are oxaliplatin, eribulin, NC1153, EPZ-6438 and R547. Oxaliplatin selectively inhibits the synthesis of deoxyribonucleic acid (DNA). Shen et al. studied the efficacy, safety and feasibility of the combination of rituximab, gemcitabine, and oxaliplatin (R-GemOx) as a first-line treatment in elderly patients with DLBCL. They found that R-GemOx might be a therapeutic option for the management of DLBCL (Shen et al., 2018).

**TABLE 2:** Potential DLBCL treatment agents.

Gene type	Drug/Small molecule	Possible effect	Evidence
Down	Formaldehyde	A metabolite of vitamin A that plays important roles in cell growth, differentiation and organogenesis acts as an inhibitor of the transcription factor Nrf2 through the activation of retinoic acid receptor alpha	DOI:10.14423/SMJ.0000000000000545
Down	Ethanol	Similar to pharmacological mTOR inhibitors, which can inhibit the mTOR signaling pathway	DOI: 10.1186/s12964-015-0091-0
Down	Dibutyl phthalate	Is expected to cause severe side effects to the central nervous system of animals and humans	DOI:10.1016/S0145-2126 (96)00033-1
Down	Paclitaxel	A synthetic macrocyclic ketone analog of the marine sponge natural product halichondrin B, which leads to the inhibition of microtubule growth in the absence of effects on microtubule shortening at microtubule plus ends	Unknown
Down	Prednisolone	Belongs to the adrenal corticotropic hormone and adrenal corticotropic hormone class and has strong anti-inflammatory effects	DOI:10.3109/10428194.2011.588761
			DOI:10.5045/kjh. 2012.47.4.293
Up	Oxaliplatin	It selectively inhibits the synthesis of deoxyribonucleic acid (DNA). The guanine and cytosine contents correlate with the degree of oxaliplatin-induced cross-linking	DOI: 10.1016/S2352-3026 (18)30054-1
Up	Eribulin	Is a microtubule inhibitor indicated for the treatment of patients with metastatic breast cancer who have previously received at least two chemotherapeutic regimens for the treatment of metastatic disease. Also being investigated for use in the treatment of advanced solid tumors	DOI: 10.1007/s00280-012-1976-x. Epub 2012 Sep 26
Up	NC1153	Specifically inhibits JAK3 via NC1153-induced apoptosis of certain leukemia/lymphoma cell lines	DOI: 10.1016/j.febslet. 2010.02.071
Up	EPZ-6438	Selectively inhibits intracellular histone H3 lysine 27 (H3K27) methylation in a concentration- and time-dependent manner in both EZH2 wild-type and mutant lymphoma cells	DOI: 10.1158/1535-7163.MCT-13-0773
Up	R547	A potent CDK inhibitor with a potent anti-proliferative effect at pharmacologically relevant doses	DOI: 10.1158/1535-7163.MCT-09-0083

## Discussion

DLBCL remains a highly heterogenous disease, with the frontline R-CHOP modality achieving only a 40–60% complete response (CR) rate in unselected patients. The prognosis of patients with DLBCL with refractory tumors or relapse remains dismal. As a result, designing more sophisticated personal treatment modalities has the potential to improve the outcomes in high-risk DLBCL patients. Although a wealth of studies has focused on targeted therapies based on the molecular classification of DLBCL, the clinical stage of DLBCL remains an important factor for choosing an appropriate treatment regime. DLBCL patients with advanced- and limited-stage disease have different responses to standard chemoimmunotherapies, due to the different genomic profiles of advanced-stage disease relative to limited-stage disease (Miao et al., 2019). In this study, we propose a new approach to gain insights into the intrinsic heterogeneity of DLBCL, which focused on comparing the transcriptomic profile of advanced- and limited-stage DLBCL and distilling the disease to a few distinctly expressed genes and hub genes that might contribute to disease progression. In general, 20 genes were up-regulated and 73 genes were down-regulated in advanced-stage samples compared to limited-stage samples. We also found that *DAB1* was negatively correlated with overall survival through survival analysis of all identified DEGs (Figure 2B, p = 0.045). Due to the limitations of differential expression analysis, it is impossible to group genes with the same function together. Therefore, we carried out weighted gene co-expression network analysis and analysis on different modules. During these analyses, 79 similar gene expression modules were found using WGCNA, among which, the thistle1 module was highly related to disease stage. KEGG pathway and GO enrichment analyses of the genes in the thistle1 module indicated that DLBCL progression was mainly related to the NOD-like receptor signaling pathway, neutrophil activation, secretory granule membrane and carboxylic acid binding. There is evidence that tumors and their mesenchymal cells produce many cytokines and chemokines to stimulate the differentiation of N2 neutrophils (Valerius et al., 1993; Souto et al., 2014). However, neutrophils can cause DNA damage through reactive oxygen species and related products of myeloperoxidase (MPO), and N2 cells secrete VEGF, TNF and other cytokines to promote tumor angiogenesis and, at the same time, synthesize and secrete MMP and NE to the tumor stroma to participate in the tumor reconstruction of the extracellular matrix to promote tumor growth and metastasis (Zvi et al., 2009; Mishalian et al., 2013; Zhou et al., 2016). During key driver analysis, 47 key drivers were identified and five hub genes were extracted from these key drivers, including *MMP1*. *MMP1* (Rosas et al., 2008) can alter the microenvironment of cells. When *MMP1* is out of balance, it accelerates the degradation of the matrix barrier and promotes the formation and growth of tumors by releasing matrix-related growth factors. Studies have shown that *MMP1* is associated with lung squamous cell carcinoma, colon cancer and adenocarcinoma.

Based on gene expression and network perturbations, 10 potential agents for the treatment of DLBCL were obtained. For instance, NC1153 can inhibit JAK3 specifically and induce the apoptosis of certain leukemia/lymphoma cell lines. Using Affymetrix microarray profiling following NC1153 treatment, Nagy et al. reported that JAK3-dependent survival modulating pathways (p53, TGF-beta, TNFR and ER stress) were altered in Kit225 cells (Nagy et al., 2010). EPZ-6438 selectively inhibited intracellular H3K27 methylation in a concentration- and time-dependent manner in both EZH2 wild-type and mutant lymphoma cells. Inhibition of H3K27 trimethylation (H3K27Me3) leads to selective cell killing of human lymphoma cell lines bearing EZH2 catalytic domain point mutations (Knutson et al., 2014).

In summary, we proposed a novel pipeline to utilize perturbed gene-expression signatures during DLBCL progression for identifying agents, and we successfully utilized this approach to generate a list of promising compounds. Whether this can be used clinically needs further research. We will continue to follow the latest developments of these agents in the treatment of DLBCL and explore its pharmaco-mechanisms under the aid of stage-of-art technologies in the future.

## Data Availability

The datasets presented in this study can be found in online repositories. The names of the repository/repositories and accession number(s) can be found in the article/Supplementary Material.
