# Supplementary figures and images for: Agent Repurposing for the Treatment of Advanced Stage Diffuse Large B-Cell Lymphoma Based on Gene Expression and Network Perturbation Analysis
Source: Front Genet. 2021 Oct 14;12:756784. doi: 10.3389/fgene.2021.756784 (PMC8551569; doi:10.3389/fgene.2021.756784)

Cluster Dendrogram

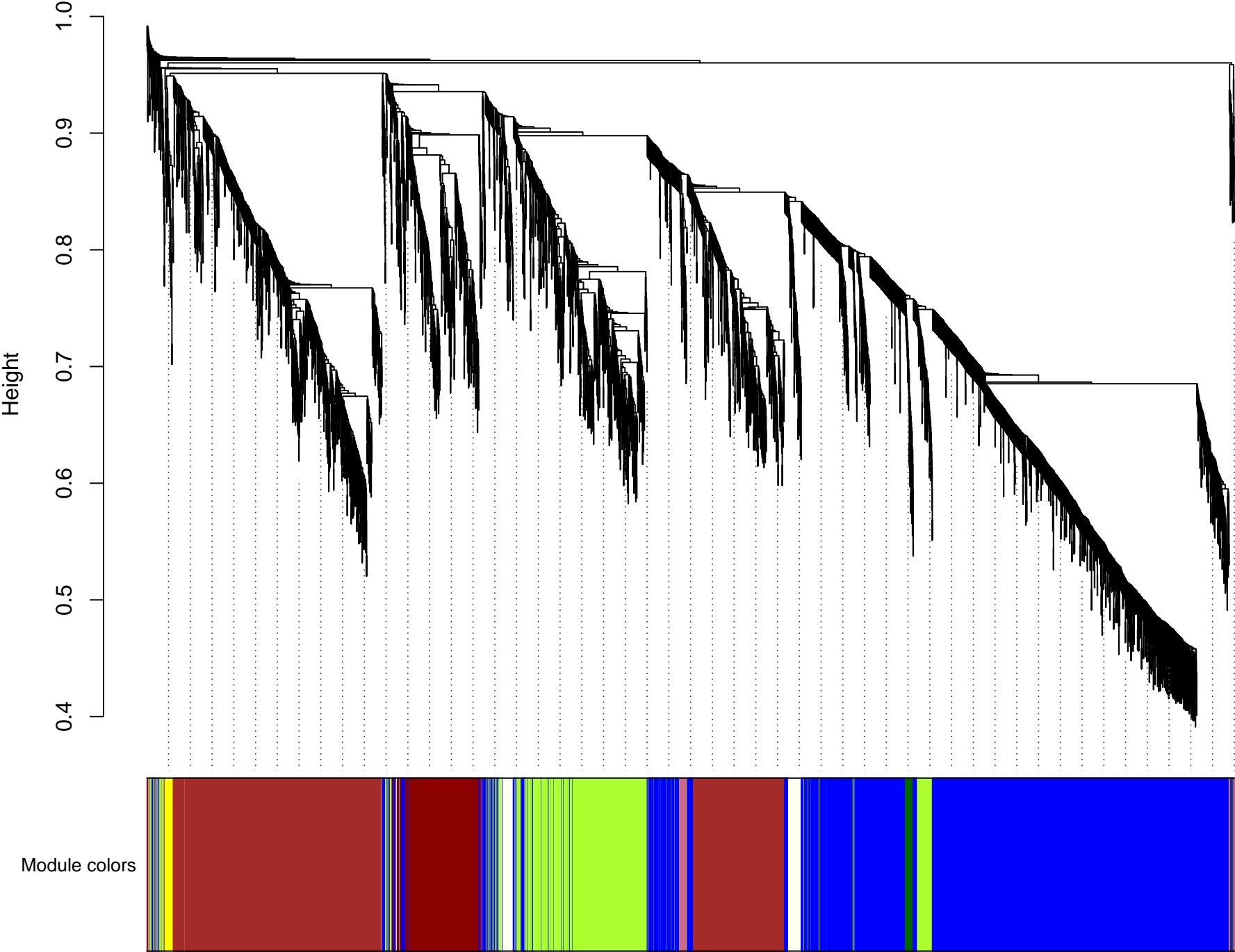

Supplement: Supplementary file 1 [file image1.pdf]
